# Adolescent and adult first time mothers' health seeking practices during pregnancy and early motherhood in Wakiso district, central Uganda

**DOI:** 10.1186/1742-4755-5-13

**Published:** 2008-12-30

**Authors:** Lynn Atuyambe, Florence Mirembe, Nazarius M Tumwesigye, Johansson Annika, Edward K Kirumira, Elisabeth Faxelid

**Affiliations:** 1 Makerere University School of Public Health, Department of Community Health and Behavioural Sciences, P.O. Box 7072, Kampala, Uganda; 2 Makerere University School of Medicine, Department of Obstetrics and Gynecology, Kampala, Uganda; 3 Makerere University School of Public Health, Department of Biostatistics and Epidemiology, Kampala, Uganda; 4 Karolinska Institutet, Department of Public Health Sciences, Division of International Health, IHCAR, SE-17177 Stockholm, Sweden; 5 Makerere University, Faculty of Social Sciences, Kampala, Uganda

## Abstract

**Background:**

Maternal health services have a potentially critical role in the improvement of reproductive health. In order to get a better understanding of adolescent mothers'needs we compared health seeking practices of first time adolescent and adult mothers during pregnancy and early motherhood in Wakiso district, Uganda.

**Methods:**

This was a cross-sectional study conducted between May and August, 2007 in Wakiso district. A total of 762 women (442 adolescents and 320 adult) were interviewed using a structured questionnaire. We calculated odds ratios with their 95% CI for antenatal and postnatal health care seeking, stigmatisation and violence experienced from parents comparing adolescents to adult first time mothers. STATA V.8 was used for data analysis.

**Results:**

Adolescent mothers were significantly more disadvantaged in terms of health care seeking for reproductive health services and faced more challenges during pregnancy and early motherhood compared to adult mothers. Adolescent mothers were more likely to have dropped out of school due to pregnancy (OR = 3.61, 95% CI: 2.40–5.44), less likely to earn a salary (OR = 0.43, 95%CI: 0.24–0.76), and more likely to attend antenatal care visits less than four times compared to adult mothers (OR = 1.52, 95%CI: 1.12–2.07). Adolescents were also more likely to experience violence from parents (OR = 2.07, 95%CI: 1.39–3.08) and to be stigmatized by the community (CI = 1.58, 95%CI: 1.09–2.59). In early motherhood, adolescent mothers were less likely to seek for second and third vaccine doses for their infants [Polio2 (OR = 0.73, 95% CI: 0.55–0.98), Polio3 (OR = 0.70: 95% CI: 0.51–0.95), DPT2 (OR = 0.71, 95% CI: 0.53–0.96), DPT3 (OR = 0.68, 95% CI: 0.50–0.92)] compared to adult mothers. These results are compelling and call for urgent adolescent focused interventions.

**Conclusion:**

Adolescents showed poorer health care seeking behaviour for themselves and their children, and experienced increased community stigmatization and violence, suggesting bigger challenges to the adolescent mothers in terms of social support. Adolescent friendly interventions such as pregnancy groups targeting to empower pregnant adolescents providing information on pregnancy, delivery and early childhood care need to be introduced and implemented.

## Background

Maternal health services have a potentially critical role in the improvement of reproductive health. The use of health services is related to availability, quality and cost of services as well as the social structures, health beliefs and personal characteristics of the users. According to the World Health Organisation (WHO), over half a million women die each year from complications of pregnancy or childbirth [1,2]. Most maternal deaths occur during childbirth and the presence of trained medical staff could greatly reduce this number [3].

During the International Conference on Population and Development (ICPD) held in Cairo in 1994, governments from around the world endorsed the need to promote and protect the rights of adolescents to reproductive health information and care [4]. However, the situation in many countries may not reflect this recognition. In some low income countries where fertility rates are high, teenage pregnancy and early marriage are common. For instance, the proportion of teenage women who are mothers or currently pregnant is highest in sub-Saharan Africa (20–40%) [5]. As a result, pregnancy and childbirth are the leading cause of death among women adolescents [6,7]. Compared to adult mothers, adolescent mothers are at increased risk of poor maternal and infant outcomes such as maternal and infant death or having an infant who is of low-birth-weight [8-10]. In sub-Saharan Africa, even when services are available, many pregnant women come late for ANC and many attend only once thereby limiting the quality of care provided [11-13]. This hampers the delivery of effective antenatal care (ANC) screening and treatment programs, potentially contributing to the high maternal morbidity and mortality [14]. Young people in particular are reluctant to seek health service for their sexual and reproductive health needs. Included among the many barriers are restrictive laws and policies [15], judgemental health workers and a lack of training in and understanding of adolescent reproductive needs [16]. There is fear among adolescents of humiliation or having to respond to unpleasant questions and procedures. Furthermore, there is lack of respect, privacy and confidentiality within the health care system [4].

The health of women is a human right issue and is a fundamental pillar for progress in low income countries. Addressing reproductive health issues is an important element in the Uganda's National Health Sector Strategic Plan (HSSP II). This plan stipulates that the utilization of maternal and child health services is still inadequate mainly because of low education and cultural practices, including power dynamics at household and community levels [17]. The Uganda demographic and health survey 2006 also established that although some improvements had been made in the area of reproductive health in the previous five years (MMR from 505 down to 435/100,000 live birth; teenage pregnancy from 31 to 25%; use of family planning from 18.6 to 24.4%; four and more ANC visits from 42 to 47%; assistance by skilled providers during birth from 39 to 42%) [18], more effort is needed in order to meet the HSSP targets and the Government poverty eradication action plan (PEAP) objectives. Adolescent pregnancy is singled out because of its association with higher morbidity and mortality for both the mother and the child. Besides, schools often terminate adolescents' education and this indirectly affects the health of the mother and the child through loss of socio-economic opportunities.

Understanding adolescents' health seeking behaviour is critical for quality service improvement. We therefore compared the health seeking practices of first time adolescents and adult mothers during pregnancy and early motherhood in Wakiso district, Uganda. This will enable us contribute towards improving reproductive health service delivery and programming for both adolescent and adult mothers in the district.

## Methods

### Study design and setting

This was a cross-sectional study conducted between May and August 2007 in Wakiso district, which has a total area of 2,704 Km^2^. The district lies in the central region of Uganda and has a population close to one million. It has two counties and one municipality, 17 sub-counties and 130 parishes with an average of four persons per household. In terms of health infrastructure the district has a total of 93 health facilities including two hospitals.

### Study population

The study population included first time mothers (adolescents aged 13–19 and adults 20–29 years) with a child less than one year. This was to ensure that pregnancy, delivery and early motherhood experiences and practices for both groups were comparable.

### Sample size and sampling procedures

Bennett's formula was used to calculate the sample size. The formula also calculates the number of clusters needed to obtain the required sample size [19]. Using Bennett's formula for cluster sampling the overall total sample size was 616 to be drawn from 88 clusters (villages) with each cluster contributing 7 respondents. However, 146 respondents were added to take care of non response. Using the 2000/01 Uganda population and housing census, we obtained a ratio of adolescents to mothers as 6:4 and we used it to get the number of adolescent and adult mothers we needed in the study. In total, 762 mothers (442 adolescents and 320 adults) were interviewed.

**Figure 1:**
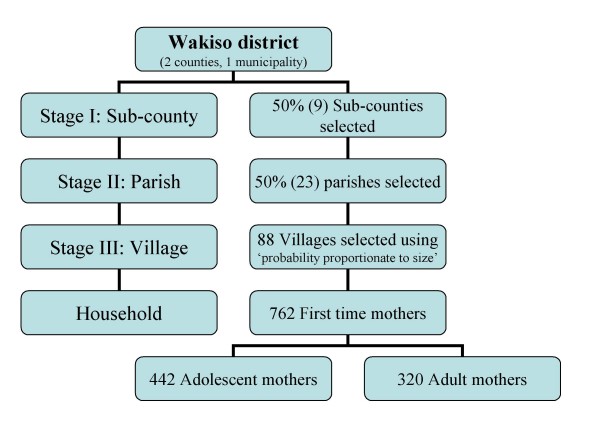
**This chart illustrates the sampling strategy**.

A list of villages and their population sizes was obtained from Uganda Bureau of Statistics (UBOS). The selected villages were visited to draw a sampling frame. During this visit, contact with the village leaders and identifying guides for the survey was made. Before data collection, letters were sent to chairpersons of the local councils with details of date of visit and a request for cooperation. Local area council chairpersons were the main point of entry in the field. A mix of multi-stage and cluster sampling techniques was applied (Figure 1).

Using multi-stage sampling method [19] 50 percent of sub-counties were randomly selected from the two counties and municipality. Thereafter, 50 percent of the parishes were randomly selected from each of the selected sub-counties. A number of villages which were proportionate to size of the parish were selected using simple random sampling from each parish. The size of the parish was determined by the number of villages. The more the number of villages in a parish, the more villages selected. The total number of villages selected was 88. In each cluster (village) we targeted to get at least 9 respondents. In each selected village we constructed two sampling frames, one comprising female adolescents 13–19 and the other adult women 20–29. Those who qualified to be on the sampling frames were first time mothers with a child not more than one year of age. About nine mothers representing nine eligible households (5 adolescent and 4 adult mothers) were randomly selected from each of the 88 villages using simple random sampling technique by using a table of random numbers. In case there was more than one eligible woman in a household, the one with the lowest last digit of their birth day (day of the month) was selected. One follow-up appointment was made in case an interview could not be carried out.

### Methods and procedure of data collection

We used a structured questionnaire with closed and a few open ended questions. Ten female graduate social scientists with experience in field data collection conducted the interviews. They all spoke the local language (Luganda) and received training for five days beforehand. The content of the training included the description of the study objectives, methods of data collection and sampling techniques. The focus of the questionnaire was on health seeking behaviour for curative and preventive services during pregnancy and early motherhood. The questionnaire had five sections, namely: general information, socio-demographic characteristics, general health status, reproductive health and child health. The training comprised a one-to-one question and answer simulation exercise including possible responses in the local language. The questionnaire was translated into the local language (Luganda) and back translated into English to ensure consistency of meaning. It was pretested on the fourth day of training in Kasangati (Nangabo sub-county) 15 km from Kampala city. After a debrief session the questions were refined. All 30 pretested questionnaires were entered in a data entry software [20] and analyzed. These questionnaires are not included in our results. Field procedures, recording and editing answers were reviewed. Written consent was obtained from respondents before interviews begun. Interviews, which lasted for an average of 45 minutes, took place in the compound often under tree shed and sometimes inside the house. Only the respondent was present during the interview.

### Quality control

Field supervisors checked for completeness, consistency and validity of every filled questionnaire before data entry. The first author (LA) conducted debrief meetings every evening to discuss daily field progress and make adjustments where necessary. Extensive validity, consistency and range checks were embedded in the data entry software by the third author (NMT).

### Data management and analysis

The filled questionnaires were entered in the computer using EPIDATA software [20]. These data were then exported to STATA V.8 for data analysis [21]. Frequency distribution of all variables was run to check for any unfamiliar pattern in the process of data entry. Odds ratios (OR) with their corresponding 95% confidence interval (CI) were calculated. All data analysis was performed using STATA V.8 [21]. Statistical significance was based on *p *value < 0.05.

### Ethical issues

Ethical approval was obtained from the higher degrees, research and ethical committees of the Faculty of Medicine Makerere University in Uganda and the regional research and ethics committee in Stockholm, Sweden. In addition, permission to conduct the study was obtained from Wakiso district local government. Written informed consent from adult mothers and emancipated minors [22] according to Ugandan ethical guidelines was sought from respondents before interviews began. Participants were informed that there were minimal or no risk to their participation in the study, that participation was voluntary, confidential and that they could withdraw their participation anytime during the interview. They were also informed that refusing to participate would not affect the usual services they normally access at health units.

## Results

### Characteristics of respondents

The mean (SD) age of adolescent mothers was 18 (1.1) years and adult mothers 22 (2.2) years. The majority of mothers (58% adolescent and 73% adult) were married or in partnership but adolescent mothers were two times more likely to be single than the adults. Thirty six percent of adult mothers belonged to the Anglican faith while a higher proportion of adolescents were Catholics and Muslims. Half of adolescents and 67 percent of adults had been to secondary school while there were significantly more adolescent mothers with no or incomplete primary education. Adolescent mothers were nearly four times more likely to have dropped out of school due to pregnancy (OR = 3.6, 95% CI: 2.40–5.44) compared to adult mothers. About half of the women in both groups, their spouses/partners provided the main source of income. About 20 percent of the adolescents and 30 percent of the adult mothers mentioned that own salary, trading and farming were the main sources of income. The former group depended more on relatives for income (Table 1).

**Table 1:** Background and socio-demographic characteristic

	**Adolescent mothers**		**Adult mothers^¥^**			
** Characteristics**	**n = 442**	**%**	**n = 320**	**%**	**OR**	**95% CI**
**Marital status**						
Single	161	(36.4)	73	(22.8)	2.01	1.44–2.79*
Divorced/separated	26	(5.9)	15	(4.7)	1.57	0.82–3.05
Married/in partnership (base outcome)	255	(57.7)	232	(72.5)		
**Education attainment**						
None/Primary incomplete	147	(33.3)	56	(17.5)	1.98	1.36–2.89*
Primary complete	76	(17.2)	50	(15.6)	1.15	0.76–1.74
Upper/post secondary school	27	(6.1)	69	(21.6)	0.30	0.18–0.48*
Secondary lower (base outcome)	192	(43.4)	145	(45.3)		
**Religion**						
Anglican	103	(23.3)	116	(36.3)	0.52	0.36–0.74*
Muslim	118	(26.7)	52	(16.3)	1.32	0.88–1.99
Other	49	(11.1)	52	(16.3)	0.55	0.35–0.87*
Catholic (base outcome)	172	(38.9)	100	(31.3)		
Dropped out school due to pregnancy**						
No (base outcome)	264	(63.9)	224	(86.5)		
Yes	149	(36.1)	35	(13.5)	3.61	2.40–5.44 *
**Main source of income**						
Salary	20	(4.5)	35	(10.9)	0.43	0.24–0.76*
Trading	48	(10.7)	48	(15.0)	0.75	0.48–1.12
Farming	24	(5.4)	18	(5.6)	1.00	0.55–1.90
Relatives	53	(12.0)	17	(5.3)	3.33	1.30–4.16*
Mother of adolescent/adult mother	21	(4.8)	6	(1.9)	2.62	1.03–6.62*
Others	38	(8.6)	18	(5.3)	1.58	0.87–2.86
Spouse/partner (base outcome)	238	(53.9)	178	(54.5)		
**Mean age (sd)**	17.9 (1.1)		22.3 (2.2)			
**Mean age of first sex (sd)**	15.9 (4.3)		18.4 (8.1)			
**Mean (sd.) age of first marriage**	16.8 (1.3)		20.1 (2. 6)			

^¥^Reference group; *statistically significant; N.B. Odds among adolescent mothers are divided by odds among adult mothers

**For only those who had been to school

### Pregnancy practices

Although the majority of adolescent and adult mothers attended ANC at least once, very few (less that 15%) attended at least once within the first three months of pregnancy. The mean number of times adolescent and adult mothers attended ANC were nearly the same (4.1 and 4.3 respectively), and adolescent mothers were 1.5 times more likely to attend less than four ANC visits (OR = 1.52, 95% CI: 1.12–2.07) compared to adult mothers. There was no difference between adolescents and adults with regard to how early they attended or where they sought ANC services. Both groups mainly attended public health institutions (Table 2).

**Table 2:** Adolescent and adult mothers pregnancy related practices

	**Adolescent mothers**		**Adult mothers^¥^**			
**Health seeking practices**	**n = 442**	(%)	**n = 320**	(%)	**OR**	**95% CI**
**ANC during pregnancy (n = 761)**						

No (base outcome)	12	(2.7)	5	(1.6)		
Yes	429	(97.3)	315	(98.4)	0.57	0.20–1.63
**Months of pregnancy at first ANC visit? (n = 744)**						
One to three months	54	(12.6)	47	(14.9)	0.82	0.54–1.25
Four and above months (Base outcome)	375	(87.4)	268	(85.1)		
**Number of ANC visits? (n = 742)**						
Less than four times	171	(40.0)	96	(30.5)	1.52	1.12–2.07*
Four and above (base outcome)	256	(60.0)	219	(69.5)		
**Mean Number of ANC visits**	4.1		4.3			
**Place for ANC (n = 729)**						
Public hospital	141	(33.8)	107	(34.3)	0.87	0.62–1.21
Private hospital/clinic	77	(18.5)	74	(23.7)	0.68	0.46–1.01
Public health centre [base outcome]	199	(47.7)	131	(42.0)		

^¥^reference group; *statistically significant; N.B. Odds among adolescent mothers are divided by odds among adult mothers

### Problems experienced during pregnancy

The women were asked about social, economic and health problems experienced during pregnancy. Almost half of both adolescents and adults said that they had experienced frequent ailments during pregnancy, such as malaria fever, swollen legs, anaemia and morning sickness. Seventeen percent of adolescent mothers said that they had lacked sufficient food during pregnancy, compared to 13 percent among the adult mothers. Significant differences between the adolescents and adults were found in terms of likelihood of being rejected by partners, victim of violence by parents and being sent away from school and/or home. Adolescent mothers were more likely to be inappropriately dressed [Self and community perception – dress no longer fitting because of pregnancy] (OR = 1.79, 95% CI: 1.29–2.49) during pregnancy and to be stigmatised by the community (OR = 1.58, 95% CI: 1.09–2.59) compared to adult mothers. Adolescent mothers were more likely to lack disposable income compared to adult mothers (OR = 1.64, 95%CI: 1.20–2.24). Up to 40 percent of mothers feared that they might die while giving birth while more than one third was not certain that their infants would survive birth. These uncertainties were significantly more common among the adolescent mothers compared to the adult mothers (Table 3).

**Table 3:** Experienced problems during pregnancy

	**Adolescent mothers**		**Adult mothers^¥^**			
***Experiences problems***	**n = 442**	**%**	**n = 320**	**%**	**OR**	**95% CI**
**Frequent ailments (n = 756)**						
No (base outcome)	235	(53.4)	168	(53.2)		
Yes	205	(46.6)	148	(46.8)	0.99	0.74–1.32
**Lack of enough food (n = 754)**						
No (base outcome)	362	(82.6)	275	(87.0)		
Yes	76	(17.4	41	(13.0)	1.41	0.93–2.12
**Violence from partner (n = 755)**						
No (base outcome)	399	(91.3)	284	(89.3)		
Yes	38	(8.7)	34	(10.7)	0.79	0.49–1.29
**Rejection by partner (n = 725)**						
No (base outcome)	358	(84.6)	272	(90.1)		
Yes	65	(15.4)	30	(9.9)	1.65	1.04–2.61*
**Violence from parents (n = 726)**						
No (base outcome)	319	(75.6)	263	(86.5)		
Yes	103	(24.4)	41	(13.5)	2.07	1.39–3.08*
**Sent away from home (n = 708)**						
No (base outcome)	369	(89.4)	289	(90.0)		
Yes	44	(10.7)	6	(2.0)	5.94	2.50–14.11*
**Expelled from school (n = 723)**						
No (base outcome)	375	(89.3)	297	(98.0)		
Yes	45	(10.7)	6	(2.0)	5.94	2.50–14.11*
**Stigmatized by community (n = 725)**						
No (base outcome)	346	(82.0)	268	(88.4)		
Yes	76	(18.0)	35	(11.6)	1.58	1.09–2.59*
**Inappropriate dressing (n = 753)**						
No (base outcome)	288	(65.8)	244	(77.5)		
Yes	150	(34.3)	71	(22.5)	1.79	1.29–2.49*
**Lack of disposable income (n = 252)**						
No (base outcome)	267	(61.1)	227	(72.1)		
Yes	170	(38.9)	88	(27.9)	1.64	1.20–2.24*
**Uncertainty of child survival (n = 755)**						
No (base outcome)	270	(61.5)	207	(65.5)		
Yes	169	(38.5)	109	(34.5)	1.19	0.88–1.61
**Uncertainty of own survive during delivery (n = 755)**						
No (base outcome)	243	(55.4)	201	(63.6)		
Yes	196	(44.6)	115	(36.4)	1.41	1.04–1.90*
**Uncertainty of continuing education after delivery (n = 737)**						
No (base outcome)	362	(84.2)	283	(92.2)		
Yes	68	(15.8)	24	(7.8)	2.22	1.36–3.61*

^¥^Reference group; *statistically significant; N.B. Odds among adolescent mothers are divided by odds among adult mothers

### Delivery practices

Over 85 percent of women were assisted by health practitioners at health units during delivery. There was no statistically significant difference between adolescents and adults with regard to where they gave birth and who assisted them. Adolescent mothers were nearly two and a half times more likely to be advised by relatives where to give birth (OR = 2.45, 95% CI: 1.21–4.91) compared to adult mothers. Around 40 percent of all mothers either walked or travelled by motorcycle when labour began. About half of the mothers indicated that lack of money was a major barrier to seeking biomedical health care (Table 4).

**Table 4:** Adolescent mothers and adult mothers delivery practices

	**Adolescent mothers**		**Adult mothers^¥^**			
**Characteristics**	**n = 442**	**%**	**n = 320**	**%**	**OR**	**95% CI**
**Assistance during delivery (n = 761)**						
Doctor	57	(12.9)	41	(12.8)	1.05	0.68–1.63
Nurse aide	31	(7.0)	18	(5.6)	1.30	0.71–2.38
TBA	19	(4.3)	18	(5.6)	0.80	0.41–1.55
Relative/friend/Others	45	(10.2)	25	(7.8)	1.36	0.81–2.28
Midwife/nurse (base outcome)	289	(65.5)	218	(68.1)		
**Place of birth (n = 758)**						
Own home	34	(7.7)	16	(5.0)	1.63	0.85–3.10
TBA's home	15	(3.4)	14	(4.4)	0.82	0.38–1.77
Public health centre	99	(22.6)	78	(24.5)	0.97	0.66–1.43
Private hospital/clinic	146	(33.3)	100	(31.3)	1.12	0.78–1.59
Public hospital (base outcome)	145	(33.0)	111	34.8)		
**Reasons for choice of delivery place (n = 746)**						
Public Hospital with all facilities	50	(11.7)	47	(14.6)	0.89	0.56–1.43
Father of child made decision	9	(2.1)	12	(3.8)	0.62	0.25–1.52
Complication requiring hospital	36	(8.4)	22	(7.0)	1.35	0.75–2.42
Free medical care	34	(7.9)	21	(6.6)	1.33	0.73–2.42
Lack of money	17	(4.0)	14	(4.4)	1.00	0.47–2.11
Referred	39	(9.1)	21	(6.7)	1.53	0.85–2.74
Advised by relative	47	(11.0)	15	(4.7)	2.58	1.37–4.85*
Others	8	(1.8)	16	(5.1)	0.41	0.17–0.99
Private hospital	18	(4.2)	13	(4.4)	1.14	0.54–2.42
Abrupt labour pain/at night	29	(6.8)	20	(6.6)	1.19	0.64–2.22
Near ANC facility (base outcome)	141	(32.9)	116	(36.7)		
**Mode of mode of transport (n = 730)**						
Commercial motorcycle/bicycles (Boda-boda)	109	(25.6)	72	(23.6)	0.96	0.90–1.01
Walking	70	(16.4)	53	(17.3)	0.96	0.90–1.04
Public transport/taxi	121	(28.4)	77	(25.3)	0.95	0.90–1.01
Others	17	(4.0)	6	(2.0)	0.87	0.76–1.01
Car/private taxi (base outcome)	109	(25.6)	97	(31.8)		
**Major barriers to health seeking for biomedical care (n = 531)**						
Fear to be found HIV positive	32	(10.3)	26	(11.9)	1.28	0.73–2.25
Rude health workers	19	(6.1)	14	(6.4)	1.16	0.56–2.40
Long distances	10	(3.2)	9	(4.1)	1.42	0.56–3.59
Prefer herbal	7	(2.2)	11	(5.2)	2.47	0.93–6.56
Others	43	(13.8)	30	(13.8)	1.10	0.75–1.84
Lack of money (base outcome)	202	(64.5)	128	(58.7)		

^¥^reference group; *significant findings; N.B. Odds among adolescent mothers are divided by odds among adults mother

### Breast feeding and supplementary feeding

Over 95 percent of women had ever breast-fed but several kinds of feeds such as sugar/glucose water, ghee in mushroom soup were given before breast milk started to flow. Adolescent mothers were more likely to delay initiation of breast feeding (OR = 1.48, 95% CI: 1.00–2.17) compared to adult mothers. They started breastfeeding after two days till breast milk flow. Most mothers (65%) introduced solid foods at six months or later (Table 5).

**Table 5:** Breastfeeding and supplementary feeding among adolescent mothers and adult mothers

	**Adolescent mothers**		**Adult mothers^¥^**			
**Variable**	**n = 442**	**%**	**n = 320**	**%**	**OR**	**95% CI**
**Ever Breastfed (n = 761)**						
No (Base outcome)	15	(3.4)	19	(5.9)		
Yes	426	(96.6)	301	(94.1)	1.79	0.87–3.58
**Start of breastfeeding after birth (n = 724)**						
Immediately (less that 1 hour)	143	(33.8)	115	(38.2)	0.96	0.68–1.34
Days (2 till breast milk started flowing)	119	(28.1)	62	(20.6)	1.48	1.00–2.17*
Hours (1 to 24) (base outcome)	161	(38.1)	124	(41.2)		
**Child given some supplements before breast milk started flowing (n = 754)**						
No	175	(40.1)	(123)	38.8		
Yes	262	(59.9)	(194)	61.2	0.95	0.71–1.28
**Supplements given before breast milk started flowing (n = 397)**						
Milk other than breast milk	21	(8.8)	20	(10.3)	0.69	0.35–1.35
Plain water	45	(17.3)	47	(24.3)	0.63	0.38–1.03
Tea infusions	32	(12.3)	28	(14.4)	0.75	0.42–1.34
Ghee/mushroom soup	37	(14.2)	17	(8.8)	1.43	0.75–2.70
Sugar/glucose water (base outcome)	125	(48.1)	82	(42.3)		
**Still breastfeeding **(at time of survey) (n = 466)						
No (outcome base)	27	(10.0)	33	(16.8)		
Yes	243	(90.0)	163	(83.2)	1.82	1.06–3.14*
**Introduction of solid foods**						
less than 6 months	86	(35.0)	74	(34.1)		
6 months and later (base outcome)	160	(65.0)	143	(65.9)	0.96	(0.65–1.40)

^¥^reference group; *significant findings; N.B. Odds among adolescent mothers are divided by odds among adult mothers

### Childhood immunization

In general, vaccination coverage (using vaccination card method) was high (69%). Adolescent mothers were significantly less likely to report possession of a vaccination card compared to adult mothers. Adolescent mothers were also less likely to seek for subsequent doses compared to adult mothers [Polio2 (OR = 0.73, 95% CI: 0.55–0.98), Polio3 (OR = 0.70: 95% CI: 0.51–0.95), DPT2 (OR = 0.71, 95% CI: 0.53–0.96), DPT3 (OR = 0.68, 95% CI: 0.50–0.92)]. However, we did not observe any difference between adolescents and adults regarding measles and Vitamin A supplementation (Table 6).

**Table 6:** Childhood immunization among adolescent mothers and adult mothers

	**Adolescent mothers**		**Adult mothers^¥^**			
**Variable**	**n = 442**	**%**	**n = 320**	**%**	**OR**	**95% CI**
**Have card where vaccinations are written**						
No (base outcome)	81	(18.4)	30	(9.4)		
Yes	360	(81.6)	288	(90.6)	0.46	0.30–0.72*
**Vaccination card seen**						
No (base outome)	106	29.8	191	33.2		
Yes	250	70.2	95	66.8	1.17	0.83–1.66
**Child vaccination for the following^€^?**						
(no = base outcome)						
BCG	251	(56.8)	190	(59.4)	0.90	0.67–1.20
Polio 0 (Polio given at birth)	205	(46.4)	161	(50.3)	0.85	0.64–1.14
Polio 1	212	(48.0)	172	(53.8)	0.79	0.59–1.06
Polio 2	172	(38.9)	149	(46.6)	0.73	0.55–0.98*
Polio 3	127	(28.7)	117	(36.6)	0.70	0.51–0.95*
DPT 1	202	(45.7)	163	(50.9)	0.81	0.61–1.18
DPT 2	155	(35.1)	138	(43.1)	0.71	0.53–0.96*
DPT 3	118	(26.7)	112	(35.0)	0.68	0.50–0.92*
Measles	60	(13.6)	54	(16.9)	0.77	0.52–1.15
Vitamin A (most recent)	63	(14.3)	49	(15.3)	0.92	0.61–1.38

^¥^reference group; *statistically significant; ^€^*data transcribed from vaccination card; N.B. Odds among adolescent mothers are divided by odds among adult mothers*

## Discussion

This study demonstrates that adolescent mothers were significantly more disadvantaged in terms of health care seeking for maternal and child health services and faced more challenges during pregnancy and early motherhood compared to adult mothers. Adolescent mothers were more likely to drop out of school due to pregnancy, less likely to earn a salary, and more likely to attend ANC fewer times compared to adult mothers. Adolescents were also more likely to experience violence from parents, to be rejected by the partner, and to be stigmatized. In early motherhood, adolescent mothers were less likely to seek for second and third vaccine doses for their infants compared to adult mothers. These results are compelling and call for urgent adolescent focused interventions.

The adolescents were less educated. They were twice more likely to have no or incomplete primary education and significantly less likely to have attended primary upper or post secondary education compared to the adult mothers. Financial constrains and low education levels have implications for the uptake of health services and how to correctly understand and implement health education messages. Adolescent pregnancy greatly affects education and we note that over one third of adolescent mothers dropped out of school due to pregnancy. Moreover, adolescents were more likely to have dropped out of school due to pregnancy compared to adult mothers. The difference could have been due to the age difference. Another possible explanation could be that currently sexual initiation starts quite early as indicated in the results. Besides, adolescents got married or involved in partnership earlier than adult mothers. There is evidence that no or low education positively correlates with early sexual activity especially among women [18]. This may imply that sexual education is still lacking or has not yet taken root and needs to be started at early age.

The majority of mothers attended ANC at least once. This proportion is comparable to the one observed in the 2006 Uganda demographic and health survey [18]. However, the majority initiate ANC late, i.e. after three months of pregnancy. According to WHO whose guidelines Uganda adopted, a pregnant woman should attend ANC at least once before 16 weeks of gestation [14,23]. Although over 60 percent attended ANC four times and above, still many women especially adolescents do not meet the minimum standard of four times. Antenatal care attendance is related to place of childbirth. According the 2006 Demographic and Health Survey, births to women who made four or more antenatal care visits are almost four times more likely to occur in a health facility than births to women who did not attend antenatal care [18]. More recently, the WHO has suggested that goal-oriented antenatal care may achieve similar health outcomes as the more rigorous schedule [23,24]. This means that each visit should include care that is appropriate to the overall condition and stage of pregnancy such as: identification of pre-existing health conditions, early detection of complications, health promotion and disease prevention, birth preparedness, and complication planning. Reasons why adolescent mothers attend ANC fewer times might be lack of operational logistics such as money for transport, low education level or the socioeconomic and gender dynamics in their homes or families as suggested in literature [25-27]. Socially, being inappropriately dressed and stigmatized makes adolescents keep away from the public [28,29]. As long as effective strategies such as to increase birth attendance by skilled personnel, provide emergence obstetric care and promote institutional deliveries remain unmet, the rate of maternal morbidity and mortality in low income countries will remain high.

Adolescents experienced several problems during pregnancy. They faced rejection and violence from partners and parents. The likely reason for rejection is that partners shun responsibility for parenthood. They feel they do not have the capacity to take on the family responsibility. Previous studies confirm that men/boys deny impregnating women, especially adolescents, because they feel they are not ready to take on the responsibility [30]. Furthermore, women, especially adolescent mothers were sent away from home and expelled from school. This could be because of reactions from parents feeling that their daughter has wasted scarce resources. This is in line with results from a qualitative study conducted earlier in the same setting [28]. In central Uganda, it is widely believed that unmarried pregnant girls should not stay in the same house as their parents, and teenage pregnancy brings shame to the family. It is a well known fact that violence affects women's health. Women experiencing violent situations are often unable to make sexual and reproductive health choices and this exposes them to many health risks as observed in the same setting [31,32]. Sadly most women remain silent about violence by an intimate partner and do not seek help [33,34]. This also has socio-psychological implications to the young pregnant mothers.

Our findings suggest that adolescent mothers faced more challenges in terms of stigma from the community because of pregnancy state and lack of disposable income. This also affected the health seeking behaviour for maternal and child health services. Furthermore, over one third of pregnant women were worried of death while giving birth and anxious about whether their child will survive. Adolescent mothers were significantly more worried of dying while giving birth compared to adult mothers. This uncertainty is probably explained by the high maternal mortality ratio and infant mortality rate (435/100,000 and 76/1,000, respectively) [18] in Uganda. Probably adolescents observe death of women and children frequently in the community. The fear of death could also be compounded by lack of support from close people such as parents and partners. Lack of information and support from the health system including health workers is another possible explanation.

Despite these challenges observed, the majority of mothers were assisted by trained health personnel during labour. Only ten percent gave birth at home or at the TBAs home compared to the 2006 UDHS where 58 percent of mothers are said to deliver at home [18]. The problem of home deliveries becomes illuminated when need for emergency obstetric care arises. Other studies too suggest that choice of place of delivery has consistently been found associated with negative maternal and neonatal outcomes [35,36]. Child birth attended by skilled personnel in a health facility is associated with lower rates of maternal and neonatal mortality and morbidity than home births [1,3]. Proximity to health facilities influenced choice of delivery place for both adolescent and adult mothers. A study conducted in rural Uganda suggested that physical access, proximity and attitude of health staff influences health seeking behaviour for maternal services [37].

Like other studies have documented in Uganda, nearly all mothers breastfed their babies though not exclusively [18,38]. However, we discovered that adolescent mothers were more likely to delay initiating breastfeeding for several days [Days ranged from 2 till breast milk started flowing] after delivery compared to adult mothers. This could possibly be a result of anxiety, inadequate support, and ignorance about breastfeeding. Besides, before breast milk started to flow, several fluids were given to the babies including tea, mushroom soup with ghee, and glucose. As suggested in the literature this practice could potentially be harmful to the health of the baby [39]. Overall, WHO encourages exclusive breastfeeding for the first six months of life and discourages unnecessary use of breast milk substitutes for those women who do not know their HIV sero-status. We postulate that there is a gap in information to young women after delivery from health providers

Vaccination practices for children are considered a good proxy indicator for health seeking behaviour in early motherhood [40]. Adolescent mothers were significantly less likely to report possession of a vaccination card for childhood diseases. We also observed that children of adolescent mothers were significantly less likely to receive the second and third doses of Poliomyelitis and Diphtheria vaccines. This might be due to lack of money for transport, social support [This refers to the support offered by the social network in terms of advice, information, counseling, financial, material and emotional support], and health education, which is usually given during ANC visits. Similar to our study, recent research done on adolescents' use of maternal and child health services in low income countries including Uganda showed that infants born to adolescents were less likely to receive vaccinations than infants born to adult mother [7].

We note that our inclusion criteria leave out mothers who could have lost their babies in infancy. This is a limitation as we cannot tell where they sought healthcare. This could partly explain the higher proportion of mothers assisted by trained health workers during delivery compared to the national statistics.

## Conclusion

This study demonstrated bigger challenges to the adolescent mothers in terms of social support, stigmatisation and violence. It also showed a high attendance to a skilled provider for maternity services. Regardless of the above, adolescents still showed poorer health seeking behaviour for themselves and their children. Adolescents made fewer ANC visits compared to adult mothers. Care of the newborn was poor for the adolescents as evidenced in the delayed initiation of breast feeding and inappropriate feeds given to the newborns before the initiation of breastfeeding. We propose comprehensive adolescent SRH programmes to reduce the severe consequences of early sexual activity. Adolescent friendly interventions such as pregnancy groups targeting empowering pregnant adolescents with information on pregnancy, delivery and early childhood care should be introduced and implemented to improve their health and that of their infants. The intriguing phenomenon of early motherhood calls for enhanced access to reproductive health services especially the never-married women. Sexual education should begin before puberty. This might offer an opportunity to girls to make informed choices about their sexual activities.

## Competing interests

The authors declare that they have no competing interests.

## Authors' contributions

LA, FM, EF, AJ & EKK contributed to the study concept and design; LA & NMT were responsible for supervision of fieldwork and the acquisition of data; data analysis and interpretation was performed by LA, NMT, FM, EF, EKK, AJ; the manuscript was drafted by LA; the manuscript was revised critically for substantial intellectual content by LA, NMT, FM, EF, EKK, AJ.
